# Estimation of Number of Pigs Taking in Feed Using Posture Filtration

**DOI:** 10.3390/s23010238

**Published:** 2022-12-26

**Authors:** Taeho Kim, Youjin Kim, Sehan Kim, Jaepil Ko

**Affiliations:** 1 Kumoh National Institute of Technology, Gumi 39177, Republic of Korea; 2 Electronics and Telecommunications Research Institute, Daejeon 34129, Republic of Korea

**Keywords:** object detection, pose classification, object mapping, feed intake measurement

## Abstract

Pork production is hugely impacted by the health and breeding of pigs. Analyzing the eating pattern of pigs helps in optimizing the supply chain management with a healthy breeding environment. Monitoring the feed intake of pigs in a barn provides information about their eating habits, behavioral patterns, and surrounding environment, which can be used for further analysis to monitor growth in pigs and eventually contribute to the quality and quantity of meat production. In this paper, we present a novel method to estimate the number of pigs taking in feed by considering the pig’s posture. In order to solve problems arising from using the pig’s posture, we propose an algorithm to match the pig’s head and the corresponding pig’s body using the major-and-minor axis of the pig detection box. In our experiment, we present the detection performance of the YOLOv5 model according to the anchor box, and then we demonstrate that the proposed method outperforms previous methods. We therefore measure the number of pigs taking in feed over a period of 24 h and the number of times pigs consume feed in a day over a period of 30 days, and observe the pig’s feed intake pattern.

## 1. Introduction

Pork is one of the most commonly available meats in the world. In the last 30 years, pork consumption in Organization for Economic Co-operation and Development (OECD) countries has increased by about 8000 tons [1]. In line with this trend, the livestock sector is increasing pork production. A pig’s feed intake depends on many factors, including temperature, social environment, health, and eating habits [2]. Feed intake at different growth stages and a feeding strategy are directly related to pig growth [3]. Voluntary feed intake of pigs affects the growth rate and feed efficiency [4]. Therefore, monitoring the feed intake of pigs in the barn will help to obtain indirect information about pig growth and the environment and increase production. On the other hand, human monitoring is labor intensive and has difficulty in providing information continuously. Radio frequency identification (RFID) systems have been used to solve this problem. However, the performance of RFID systems depends on the location of the antenna [5]. They not only are environmentally sensitive but also take a lot of time and money because they have to be attached to many pigs [6].

Advances in artificial intelligence have allowed us to extract useful information from images. Some studies have applied this to the livestock sector. Zhang et al. proposed a system for pig detection and tracking under various environmental conditions [7]. Tian et al. proposed a system for counting pigs in the barn based on the ResNeXt model [8]. Shi et al. proposed a system that measures pig body information by inputting 2D and 3D images [9]. Ahn et al. proposed a system for estimating pig size based on segmentation. They counted pixels excluding the head region for stability [10]. Chen et al. acquired motion information through the detected position of the pig. This was used to detect aggressive behavior in pigs [11,12]. Li et al. studied a system based on Mask-RCNN to detect a pig’s mounting behavior [13]. Marsot studied pig face recognition based on a CNN model [14]. Recently, in [15], the authors conducted research on the detection of individual pigs in a barn and used a variation of YOLOv3 to obtain high accuracy for the detection of pigs. The research neither contains the pose estimation of pigs nor counts the pigs that are actively feeding.

Some studies have raised detection performance or analyzed behavior through animal posture information. Riekert et al. studied object recognition technology that accurately identifies the location of pigs in various barn environments. They showed that the detection performance can be improved by classifying the pig’s posture information into a separate class [16]. Nasirahmadi et al. proposed a system to detect standing and lying pigs separately. They analyzed the pigs’ group activity through the ratio of their standing over time [17]. Li et al. organized the system by distinguishing five postures [18]. Shao et al. proposed a system for classifying pig posture based on semantic segmentation [19]. Lao et al. studied the classification of postures in 3D images [20]. These studies raised detection performance and analyzed behavior through pig posture. However, they did not consider feed intake information or count accurately.

It is also possible to measure feed intake using AI-based technology. To determine the feed intake of pigs, Yang et al. marked pigs with the letters A through D. Feed intake was determined through the intersection area between the head and the feeder [21]. It is interesting to note that pigs were marked with alphabets to distinguish them. However, it is difficult to use such a system in big pig barns. Alameer et al. installed a camera just above the feeding trough. Because the camera was close to the feeding trough, it can capture up to four pigs. Therefore, they divided the images into seven classes: no pigs, one pig, one eating, and so on [22]. The methodology achieved over 99.4% accuracy on the collected data set and a faster processing speed of 50 fps. However, this system requires a separate camera close to the feeding trough. Kim et al. [23] detected pigs by YOLOv3, v4 models. In the paper, whether to consume or not to consume feed was designated as a class while detecting the pigs. Additionally, unlike other methods that used such object detection models, this paper does not perform any postprocessing, such as IoU (Intersection over Union) calculations. Although it demonstrated fast processing speed and high accuracy in the YOLO family of object detection models, when pigs in the images overlapped, their performance became relatively poor, and they could not perform for unusual cases like those in different postures, such as sleeping near the trough.

It is seen that most of the times, the research topic is focused on detecting pigs rather than detecting pigs that are feeding in a barn. In [21,22,23], the authors provide a way to detect pigs that are feeding by applying various novel methods. However, even then, they failed to successfully identify the pigs that were feeding and distinguish the ones that were not. Additionally, they did not use pig posture information. Several false-positive cases were observed, where a pig would just lie near the trough and not actually feed but be classified under pigs that were feeding. Our paper addresses such problem and compares the results with existing related papers.

Our contribution in this paper includes detection of pigs who are feeding using a novel approach. After the candidate pigs are chosen using distance estimation from the trough, we map the heads of the pigs with their respective bodies. This helps us in establishing the foundation for posture filtration, where we filter out nonfeeding pigs from the candidate set based on the detected posture. We compare our results of detected feeding pigs with the methods proposed by [21,22,23]. We then perform a brief analysis on the eating habits and behavioral patterns of the pigs in consideration.

Section 2 introduces the data set and the proposed method. In Section 3, we compare the performance of the detection model according to the anchor box and confirm the detection performance. We also compare the number of pigs taking in feed in the image with previous methods. Finally, we show the behavioral patterns by performing calculations over a span of time. In Section 4, we discuss the proposed method and draw some valuable conclusions.

## 2. Materials and Methods

### 2.1. Dataset

**Figure 1:**
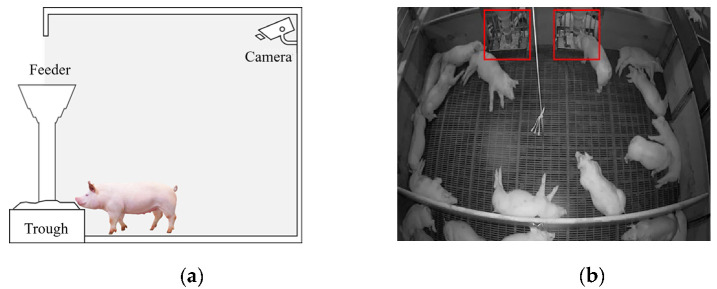
(**a**) The location of the camera installation and trough in the pig barn; (**b**) an example of an image taken in a pig barn.

The pig barn in the collected dataset has two food tanks and imaging equipment installed at a fixed location at the top of the wall. The imaging apparatus provides a fixed top-view image [24]. Figure 1a is a plain view of the location of the camera installed in the pig barn and the location of the food tank, and Figure 1b is an example of an image collected from the installed camera. The red anchor box shows the fixed positions of the trough in the pig barn in an image.

There are a total of 911 RGB images of the pixel size 480 × 704 obtained at 1 h intervals over 37 days in a pig barn. The images from the barn appear grayscale to the human eye because of the lighting conditions. Even during the daytime, there is not enough supporting light to obtain a bright color image [24]. The camera configuration in each image is consistent and shows a similar view containing two troughs each. Images were captured on an hourly basis for a period of a little over a month. The obtained images were resized to 640 × 640 and were normalized before proceeding into the next phase. The dataset for the object detection model was divided into training, validation, and test sets at a ratio of 8:1:1. Table 1 shows the number of images and classes for each dataset.

The dataset contained four classes—pig’s Head, Standing, LyingBelly, and LyingSide postures along with their respective anchor boxes. We manually added two more classes that were needed for our research. We added the anchor boxes for the trough locations, which were used to feed the pigs and another class to indicate the pigs that were feeding.

### 2.2. Problem of the Previous Method

To determine the set of pigs that were feeding, the distance between the pig and the trough was taken into consideration. The proximity between the two was calculated by using the intersection area of the pig and the trough [22]. However, it was not enough to determine whether the pigs were actually feeding. For instance, the pigs could just stand or lie idle near troughs instead of feeding. This raised a few exceptions for which we propose a novel method for the detection of pigs that are (actually) feeding.

### 2.3. Proposed Method

**Figure 2:**
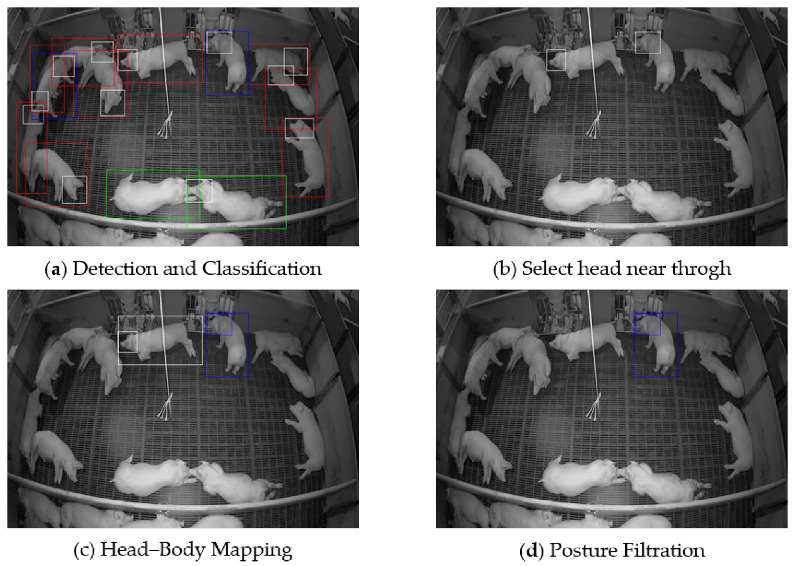
Shows the output after different methods in the system. (**a**) shows the detection and classification output of YOLOv5. White boxes denote head positions, red boxes indicate the pigs lying on their sides, green boxes denote the pigs lying on their bellies, and blue boxes denote the standing pig. (**b**) shows the output of candidate pigs whose head is close to the trough. (**c**) shows the output of the pigs after head and body mapping. (**d**) shows the final output after posture filtration.

**Figure 3:**
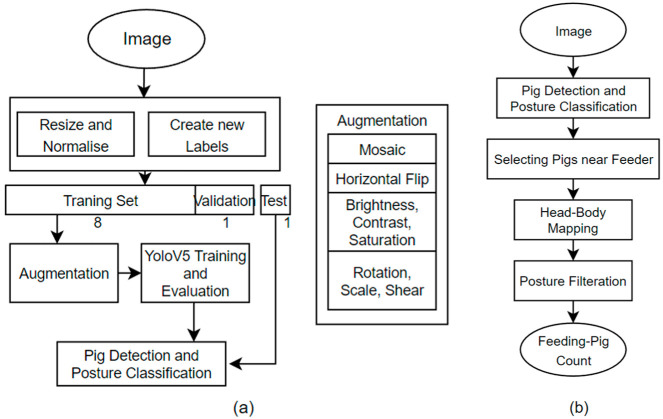
(**a**) represents the flowchart of the training model part of the proposed method, and (**b**) represents the postprocessing and inference part of the proposed method.

Our research proceeds as follows: First, object detection is performed to detect pigs in the images. Through this process, we generate various features that can be correlated with different body parts of pigs. However, for our research purposes, we only consider two features, which are head and body. The head and body of the pig follows a one-to-one correspondence where the detected head and body pair belong to a single pig. This one-to-one correspondent pair is later helpful in asserting whether or not the pig is feeding. Then, from the recognized set of pigs, candidates closer to the trough are selected for further analysis. Finally, posture filtration is performed on the set to filter the final set of pigs who are deemed to be feeding using the detected posture on the head–body paired pigs. Figure 2 shows the output flow after different steps of the system. A flowchart of the proposed system is shown in Figure 3.

#### 2.3.1. Pig Detection and Posture Classification

The initial step is to pass the image through an object detection algorithm to detect the pigs in the image. For the purpose of this research, we chose YOLOv5. This process helps to learn the features in the images that correspond to the head and body of the pigs, their location in the image, and the location of the trough. Apart from that, while detecting the body, our model classifies the pig’s postures, that is, whether it is standing, LyingBelly, or LyingSide. Standing is considered a position in which pigs eat while being supported by their four legs. LyingBelly is lying with the belly on the floor, and LyingSide is lying sideways.

One of the methods for improving the performance of the object detection model using the anchor box is appropriately initializing the anchor box. Fixing the aspect ratio and size of the initial anchor box improves accuracy when training an object detection model. This can be obtained by performing clustering on the correct target class label of the collected data set. The clustering algorithm uses the K-means algorithm and the evolutionary algorithm to set the coordinates of the anchor boxes. We discuss further the selection of anchor box configuration in Section 3.1.1.

#### 2.3.2. Selection of Pigs near the Feeder

After obtaining the position of the feeder and the position of the pigs, it is essential to shortlist the pigs that are close to the trough. In order to do so, we calculate the intersection of the area of the feeder with the area of the detected pig, as shown in Equation (1). A threshold value is used along with the intersection area to find out the pigs that are closer to the feeder bin and possibly feeding.

(1) $$\frac{Area\;of\;Pig\;Head\;\cap Area\;of\;Feeder}{Area\;of\;Pig\;Head\;\cup Area\;of\;Feeder}$$

**Figure 4:**
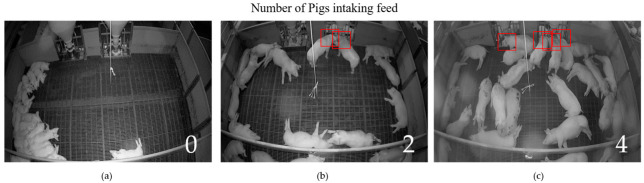
Represents the candidate pigs whose heads are close to the trough. Each (**a**–**c**) represent 0, 2, and 4 pig heads detected by the algorithm in the pictures.

It is inferred that if the value is less than the threshold, then the pig is far away from the trough and should not be considered for further analysis. On the other hand, if the value is more than the threshold, then the pig is added to the list of candidate pigs and is subjected to further analysis. Figure 4 shows the pig’s head detected by the algorithm in the picture.

**Figure 5:**
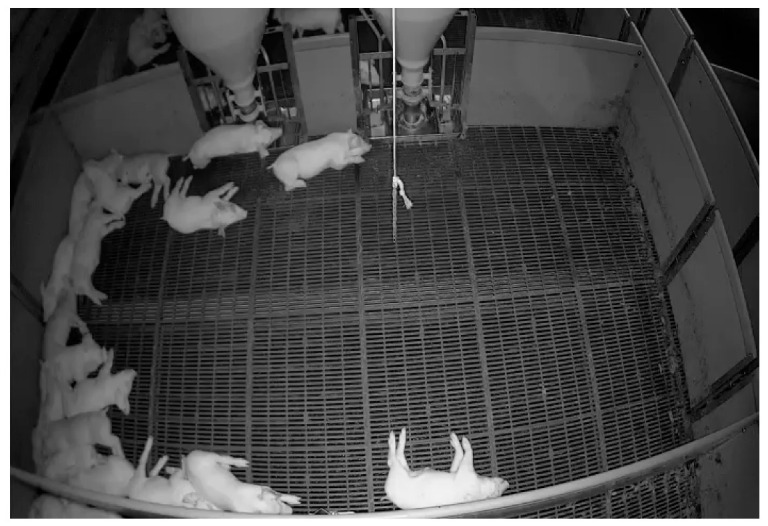
Shows pictures of pigs present near the trough but just lying idly without feeding.

The pigs, thus, obtained from the above selection indicate those that are close enough to the feeder are to be considered feeding. However, as shown in Figure 5, there are many instances where the pigs are near the feeder but are not eating.

As can be seen from the images, it is difficult to judge whether the pig is eating just by the positions of the pig’s head and the trough. To infer whether the pig is feeding, we propose to consider both the pig’s head and body and then analyze their postures.

#### 2.3.3. Body–Head Mapping Algorithm

When considering the pig’s body, it is of paramount importance that we associate the correct head with the correct body of the pig in a one-to-one correspondence. That is, the head and body pair should belong to a single pig.

Mapping is performed by calculating the intersection area between the head and the body. Since the size of the pig varies from one pig to the other, the intersection area is divided by the size of the pig head and is normalized. Equation (2) is used to perform this calculation.

(2) $$\frac{head\;of\;pig\;\cap body\;of\;pig}{head\;of\;pig}$$

When this value exceeds a specified threshold, it is referenced that the pig’s head is covered by other pigs or is not fully visible; in such cases, the reference is made based on its body. The head and body mapping of the pigs is shown Algorithm 1.
**Algorithm 1** Head-Body Mapping1:**Input**2:
H–Head object3:
B–Body object4:**Output**5:
P–Mapped Head Body Pair6:**Procedure**7:
P ← Empty list8:
for *i* ← 1 to length of H do9:

head ← H[*i*]10:

if head = NULL then11:


continue12:

else13:


temp ← Empty list14:


for *j* ← 1 to length of B do15:



body ← B[*j*]16:



if body = NULL then17:




continue18:



else if IoU(head, body) > 0 then19:




temp.push(*j*)20:


*j* ← the body with the greatest distance between center points21:


body ← B[*j*]22:


B[*j*] ← NULL23:


P.push(Pair(head, body))24:
return P

**Figure 6:**
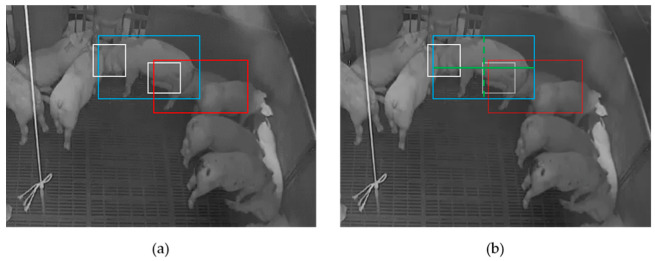
(**a**) shows two pigs colored in blue and red. The heads are drawn with white color. (**b**) shows the major axis in a green solid line and the minor axis in a dotted line on the same figure as (**a**). The head farthest from the center of the major axis is chosen as the head of the pig.

However, there are cases where it is difficult to respond only to the degree of overlap of detected objects. In Figure 6a, the boxes drawn with red and blue lines represent the degrees of overlap between two different pigs. As can be seen in the picture, the heads and bodies of two different pigs are very close to each other. In order to correspond a head with its appropriate body, we propose to draw major and minor axes in the bounding boxes. In Figure 6b, the major and minor axes of the pig are drawn with a green line. The head at the major axis end of the body is assigned to the pig’s head. In other words, the head with the farthest distance from the center point among the overlapping heads is chosen as the head for the corresponding body.

#### 2.3.4. Posture Filtration

After the head and body pair is made, we can now move to filtering the posture of the pigs so that we can eliminate the pigs that are not feeding. While training, our model also outputs the posture of the pigs. This helps us in filtering out pigs that are idly lying down and filtering the ones that are not feeding. Posture filtration is presented in Algorithm 2.
**Algorithm 2** Posture Filtration1:**Input**2:
P–Mapped Head-Body Pair3:**Output**4:
result–Number of pigs (int)5:**Procedure**6:
for *i* ← 1 to length of P do7:

head, body ← P[*i*]8:

if body! = standing then9:


P[i] ← NULL10:
result ← the number of non-null pairs in P11:
return result

**Figure 7:**
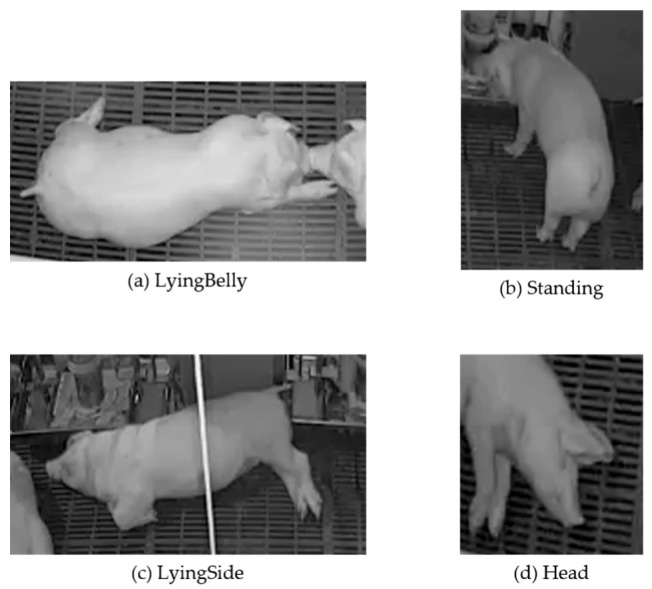
List of available postures.

Due to the height of the trough, it was observed that the pigs always ate while standing on four legs. Additionally, the ones that were not feeding were lying on the floor. Therefore, three classes were created while model-training, which consisted of Standing, LyingBelly, and LyingSide. The LyingBelly and LyingSide postures are considered to be difficult for pigs to eat from the trough. Figure 7a–c shows examples of possible posture filter classes. In addition, Figure 7d is an example of a class for pig heads.

From the above process, we try to predict the number of pigs eating at any given time. However, we also need to address the way in which our research correlates in determining the eating habits and behavioral patterns in pigs, which we discuss in a latter section.

## 3. Results

In this section, we will discuss the experiment parameters and results. The experiments are organized in the following several parts, including the process of anchor box selection, output of the detection and classifier model, quantitative analysis where our result is compared with models proposed in related works, and qualitative analysis where we reason the findings from our research and their usefulness.

The experiments were conducted with the CPU AMD Ryzen 7 5800X 8 Core Processor, RAM 32 GB, and GPU NVIDIA RTX A6000 hardware specifications. The operating system was Ubuntu 20.04. The programming language was Python 3.9.

### 3.1. Detection and Classification Model

We used the YOLOv5m model as an object detector. The optimizer was Adam W, the momentum was set to 0.937, and the learning rate was set to 0.01.

#### 3.1.1. Anchor Box Selection

While YOLO models can predict bounding boxes, the accuracy still largely depends on the correctly defined anchor boxes in the training set. Anchor boxes are the bounding boxes having a certain height and width that are defined to capture the classes while maintaining some scale and aspect ratio [25]. Moreover, the auto-anchor feature was added to YOLOv5, where the model checks for the correctness of the provided anchor boxes and recalculates them if necessary [26]. Taking this information into account, we performed some experiments to find the right anchor box configuration.

**Figure 8:**
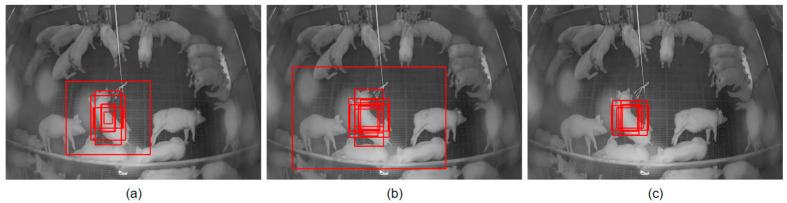
(**a**) shows the anchor boxes when trained on a default anchor box configuration, (**b**) shows anchor boxes when trained on an auto-anchor box with K set to 9, and (**c**) shows anchor boxes when trained on an auto-anchor box with K set to 6.

Table 2 draws a comparison between three different anchor box configurations. Table 2a denotes a case where the model uses the default anchor box configuration where it does not change the anchor boxes given during training but displaces them to have a better prediction. The default configuration enables the model to output 9t anchor boxes. In Table 2b, the auto-anchor box is used with a K value (used for K-means clustering) set to 9. This was performed in order to check the output when the anchor boxes were reinitialized based on the training data properties. Table 2c shows the output when auto-anchor box was used with a K value of 6. It was observed that the output of the default anchor box configuration outperformed the auto-anchor box configurations. From this, we infer that the anchor box initialization was performed in the right way, and it performed better than auto-anchor box configurations. Figure 8 shows the above observation on an image captured in the pig barn. Figure 8a corresponds to the default anchor box settings and learns from the data provided in the training set. Figure 8b shows the auto-anchor box with the K value set to 9. The generated boxes do not accurately define the pig in consideration. Figure 8c shows when the auto-anchor box setting was used with the K value set to 6. The model performs well but is very stringent in drawing the bounding boxes. After observing the results, we decided to not use the auto-anchor box setting and use the default one, where the model learned the bounding boxes from the information provided in the training set.

#### 3.1.2. Training of Detection and Classification Model

**Figure 9:**
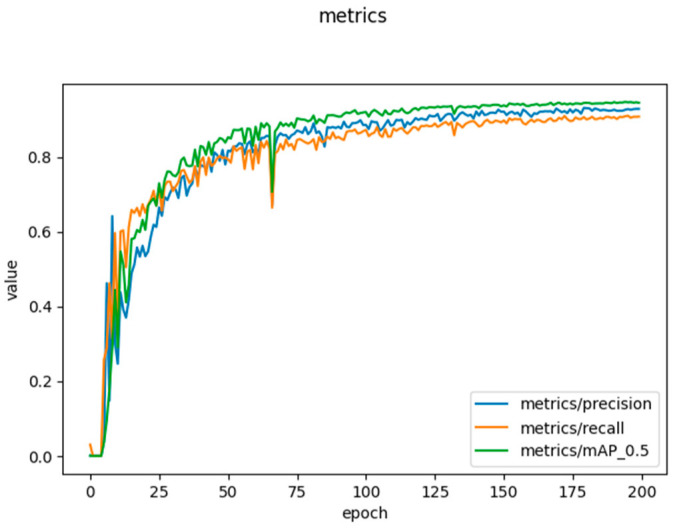
Shows the validation metrics output by the YOLOv5 model containing precision, recall, and mAP.

The performance of the learned object detection and classification model achieved a mAP of 0.942, a precision of 0.93, a recall of 0.91, and a confidence of 0.5. Figure 9 plots the model metrics output, where precision, recall, and mAP are measured with the validation dataset.

**Figure 10:**
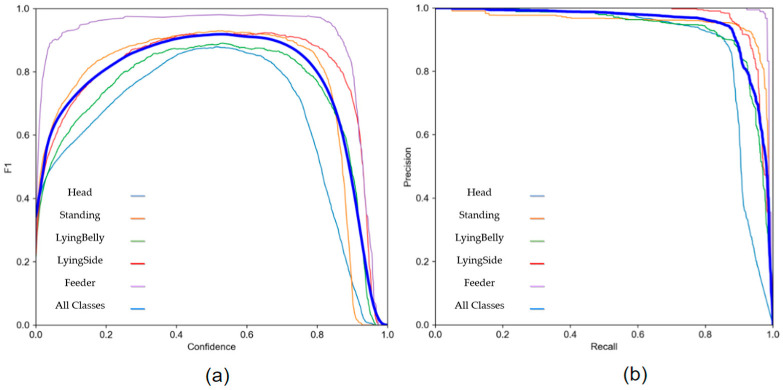
(**a**) shows the confidence Vs’ F1 graph, while (**b**) shows a recall vs. precision graph of the YOLOv5 model in validation. The classes present in the graph include Head, Standing, LyingBelly, LyingSide, Feeder, All Classes.

Precision is the fraction of instances among the retrieved instances, while recall denotes the fraction of relevant instances that were retrieved. Figure 10a plots a graph between the harmonic mean of precision and recall also known as F1 with confidence and outputs an F1 score of 0.92 at 0.532 confidence. Figure 10b plots between precision and recall with a mAP of 0.5.

### 3.2. Quantitative Analysis

Accuracy was calculated for test images to quantitatively analyze the performance of the system. We represent error as the absolute value of the difference between the number of feed intakes and the predicted feed intake present in the image. The sum of absolute difference ratio (SADR) is defined as the sum of the errors present in the image divided by the total population, as shown in Equation (3):

(3) $$\mathrm{SADR}=\underset{i}{\sum }\frac{\left|G_{i}-P_{i}\right|}{T}$$

where P_i_ and G_i_ are actual and estimated number of pigs taking in feed in the given image I, and T is the number of pigs in a barn. SADR indicates that the model detects the whole *pig* that is actually eating, calculates the difference between the predicted number of pigs and the actual pigs, and then normalizes them based on the total number of pigs in the barn.

We performed SADR calculation on our paper as well as some of the previously mentioned papers. Table 3 depicts the same, and it is noted that a lower SADR value gives a better result. It was seen that performing postprocessing methods on the images yielded a better result compared with when not using any additional methods. Kim et al. performed poorly on our dataset as it was unable to detect pigs that were in close proximity to each other and was unable to identify those that had different postures, such as sleeping near the trough. Alameer et al. showed a comparatively better performance because of a higher number of classes in the classification of pig postures in the image. Yang et al. obtained a value of 2.98 as it used a postprocessing method of distance estimation (DE) between the pig and the trough and only considered the pigs that passed the distance estimation threshold. Our proposed method also uses distance estimation along with posture filtration to achieve an even lower SADR value.

### 3.3. Qualitative Analysis

**Figure 11:**
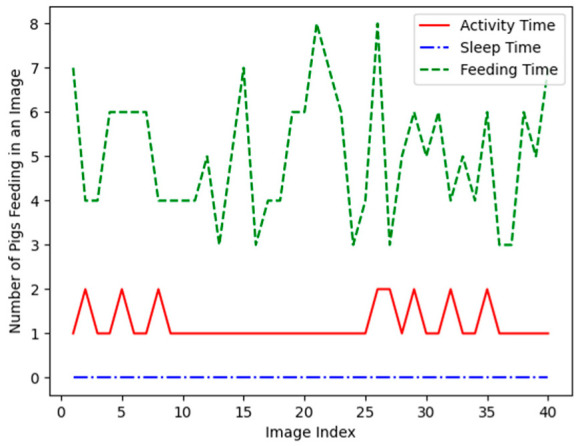
Shows the number of pigs that are eating feed in images that are captured at a certain time frame.

Pigs show different patterns of behaviors, such as gathering near the food tank depending on the time, actively wandering around, or sleeping around the corner. Figure 11 is a graph measuring the number of pigs that are feeding in a set of images captured at a certain time. The solid red line is the time when the pigs are actively moving, and it can be seen that one or two pigs are feeding. The blue dash-dotted line represents the time to sleep, and it can be seen that none of the pigs are feeding. The solid green line shows that the pigs actively feed during mealtime.

**Figure 12:**
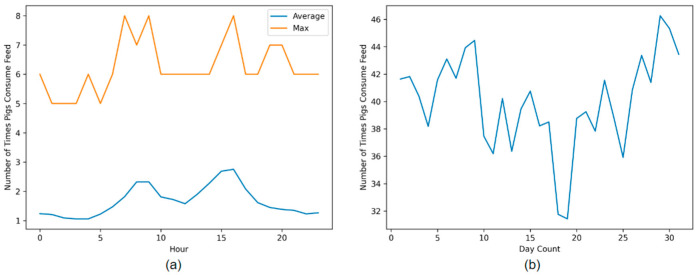
(**a**) shows the number of times pigs consume feed over a period of 24 h. (**b**) shows the number of times pigs consume feed in a day over a period of 30 days.

Furthermore, Figure 12a shows the number of times pigs are eating throughout the whole day. It can be observed that there is a spike in the morning and in the afternoon, which gives insights into the feeding patterns of the pigs in the barn. The appearance of such pattern in feed intake during the afternoon and the night behavior corroborates what has been said in previous studies [27]. Figure 12b shows the average number of times the pigs are feeding in a day for over 30 days. Such analysis over a substantial period can give useful information regarding behavior and feed intake, which can help in deriving a relationship between feed efficiency and growth rate among pigs [28].

Studies on behaviors of barn animals have indicated that illness causes animals to spend more time resting and less time feeding [29]. The feeding and ruminating behavior of barn animals provides vital information about their health, welfare, and productivity [30]. Therefore, alterations in feeding behavior could also be indicative of illness in barn animals and, in our case, pigs.

## 4. Discussion and Conclusions

This paper presents a novel approach to detecting pigs that are actually feeding using YOLOv5 for object detection along with pig head–body mapping and posture filtration. The object detection model enables identifying the pig’s head, body, their landmarks, trough location, pig’s posture, and whether the pig is feeding. Using this output information, candidate pigs are selected through distance estimation between the trough and the pig’s head. Mapping the pig’s head and body helps in identifying the pig as a whole unit, which is then used for posture filtration, where the pigs that are not standing are filtered out. Such an approach eliminates unusual cases of pigs that are lying near the trough and not feeding. In addition, quantitative and qualitative analyses are performed, where we compare the SADR score of our model with the related papers in the field. It was seen that our proposed method scored the lowest SADR value, which highlights our approach of implementing pose filtration for calculating the number of pigs feeding in any given picture. Useful information regarding the health and feeding habits of pigs can be known, which becomes influential in monitoring growth among pigs. Further research can be performed to increase the performance of the model by giving special attention to the posture classification and overlapping of pigs near the trough. Through this paper, we proposed a practical method of estimating the number of pigs feeding at any given time in a picture by using the YOLO model to detect pigs in the image and then performing postprocessing of distance estimation and posture filtration in livestock barns. The identification and management of pig growth through the measurement of feeding-pig count can be influential in the proper management, nurturing, and breeding of pigs.

## Figures and Tables

**Table 1:** Number of images and classes per data set.

Classes	Dataset		
	Train	Valid	Test
Images	734	90	97
Head	14,095	1732	1683
Standing	5835	728	688
LyingSide	4442	547	534
LyingBelly	3817	456	447

**Table 2:** Draws a comparison between the performance of different anchor box configurations. (a) shows the results of the default anchor box configuration, (b) shows an auto-anchor box configuration with the K value set to 9, and (c) shows an auto-anchor box configuration with the K value set to 6. P represents precision, R represents recall, and mAP represents mean average precision.

Class	Images	Instances	P	R	mAP50	mAP50-95
All	89	3569	0.929	0.906	**0.943**	**0.657**
Head	89	1690	0.917	0.852	0.895	0.477
Standing	89	702	0.937	0.926	0.951	0.627
LyingBelly	89	537	0.871	0.866	0.915	0.658
LyingSide	89	464	0.929	0.903	0.962	0.749
Feeder	89	176	0.992	0.983	0.99	0.773
(**a**)						
All	89	3569	0.92	0.893	**0.936**	**0.648**
Head	89	1690	0.922	0.852	0.882	0.459
Standing	89	702	0.93	0.926	0.953	0.626
LyingBelly	89	537	0.847	0.866	0.905	0.656
LyingSide	89	464	0.918	0.903	0.955	0.757
Feeder	89	176	0.98	0.983	0.984	0.743
(**b**)						
All	89	3569	0.925	0.889	**0.934**	**0.647**
Head	89	1690	0.919	0.826	0.88	0.462
Standing	89	702	0.934	0.907	0.945	0.624
LyingBelly	89	537	0.857	0.846	0.914	0.655
LyingSide	89	464	0.933	0.896	0.952	0.742
Feeder	89	176	0.982	0.972	0.976	0.751
(**c**)						

**Table 3:** Performance analysis of various related models where DE denotes distance estimation and PF denotes posture filtration.

Methods	Remarks	SADR
Kim et al. [23]	No postprocessing	12.9
Alameer et al. [22]	No postprocessing	3.92
Yang et al. [21]	DE	2.98
Proposed method (ours)	DE + PF	**1.30**
